# Structures of *Leishmania* Fructose-1,6-Bisphosphatase Reveal Species-Specific Differences in the Mechanism of Allosteric Inhibition

**DOI:** 10.1016/j.jmb.2017.08.010

**Published:** 2017-10-13

**Authors:** Meng Yuan, Montserrat G. Vásquez-Valdivieso, Iain W. McNae, Paul A.M. Michels, Linda A. Fothergill-Gilmore, Malcolm D. Walkinshaw

**Affiliations:** Centre for Translational and Chemical Biology, School of Biological Sciences, University of Edinburgh, Michael Swann Building, Max Born Crescent, Edinburgh EH9 3BF, UK

**Keywords:** F16BP, fructose 1,6-bisphosphate, F6P, fructose 6-phosphate, FBPase, fructose-1,6-bisphosphatase, G6PDH, glucose-6-phosphate dehydrogenase, *h*lFBPase, human liver fructose-1,6-bisphosphatase, *h*mFBPase, human muscle fructose-1,6-bisphosphatase, *Lm*FBPase, *Leishmania* fructose-1,6-bisphosphatase, PGI, phosphoglucose isomerase, Pi, phosphate, *p*lFBPase, pig liver fructose-1,6-bisphosphatase, TDA, thermal denaturation assay, TEA, triethanolamine, TLS, translation libration screw, enzyme kinetics, crystal structure, allostery, gluconeogenesis, leishmaniasis

## Abstract

The gluconeogenic enzyme fructose-1,6-bisphosphatase has been proposed as a potential drug target against *Leishmania* parasites that cause up to 20,000–30,000 deaths annually. A comparison of three crystal structures of *Leishmania major* fructose-1,6-bisphosphatase (*Lm*FBPase) along with enzyme kinetic data show how AMP acts as an allosteric inhibitor and provides insight into its metal-dependent reaction mechanism. The crystal structure of the apoenzyme form of *Lm*FBPase is a homotetramer in which the dimer of dimers adopts a planar conformation with disordered “dynamic loops”. The structure of *Lm*FBPase, complexed with manganese and its catalytic product phosphate, shows the dynamic loops locked into the active sites. A third crystal structure of *Lm*FBPase complexed with its allosteric inhibitor AMP shows an inactive form of the tetramer, in which the dimer pairs are rotated by 18° relative to each other. The three structures suggest an allosteric mechanism in which AMP binding triggers a rearrangement of hydrogen bonds across the large and small interfaces. Retraction of the “effector loop” required for AMP binding releases the side chain of His23 from the dimer–dimer interface. This is coupled with a flip of the side chain of Arg48 which ties down the key catalytic dynamic loop in a disengaged conformation and also locks the tetramer in an inactive rotated T-state. The structure of the effector site of *Lm*FBPase shows different structural features compared with human FBPases, thereby offering a potential and species-specific drug target.

## Highlights

• *Leishmania* fructose-1,6-bisphosphatase provides a potential drug target.
• *Lm*FBPase co-crystal structure with AMP reveals its allosteric inhibitory mechanism.
• The allosteric *Lm*FBPase is regulated by a species-specific effector loop.
• AMP unlocks interface bonds to induce dimer rotation to the *Lm*FBPase inactive form.
• The poorly conserved *Lm*FBPase AMP site offers a species-specific druggable pocket.

## Graphical abstract

**Figure:**
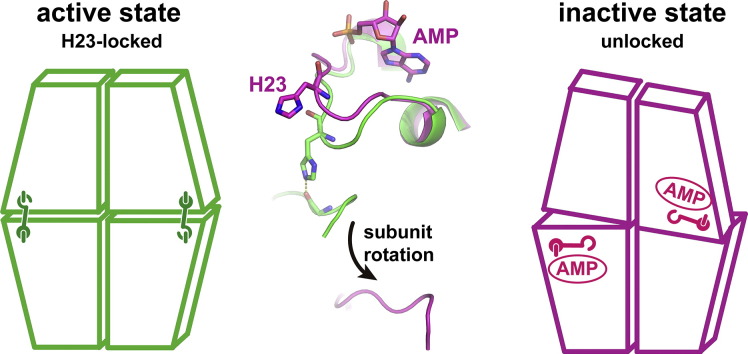

## Introduction

Trypanosomatids of the genera *Trypanosoma* and *Leishmania* are parasitic protists that cause a spectrum of diseases in humans and many animals. In particular, *Leishmania* species are responsible for cutaneous diseases (ulcers and destructive lesions) and for a visceral form involving an infection of the liver and spleen that is usually fatal if untreated. The World Health Organization estimates that approximately 700,000 to 1 million new cases of leishmaniases occur annually and are prevalent in 98 countries on five continents [1]. Of these cases, 300,000 are the visceral form also known as kala-azar, and estimates of deaths from this disease range from 20,000 to 30,000 people annually [2]. *Leishmania* exist in two main morphologically distinct forms: motile flagellated promastigotes, which occur in the sand-fly vector and are injected into human skin. After phagocytosis by macrophages, they develop into non-flagellated amastigotes in the macrophage's phagolysosome where they proliferate. Anti-leishmanial vaccines are not currently available, and treatments rely on chemotherapy but are characterized by low efficacy, toxicity, and/or widespread resistance [3], [4], [5].

The gluconeogenic pathway of *Leishmania* converts metabolites into sugar phosphates to be used in the pentose phosphate pathway for synthesis of mannogen and glycoconjugates, which are essential for amastigote replication and virulence [6]. Fructose-1,6-bisphosphatase (FBPase) is a gluconeogenic enzyme that catalyzes the transformation of fructose 1,6-bisphosphate (F16BP) to fructose 6-phosphate (F6P) and phosphate. It is found in glycosomes, organelles related to peroxisomes, and found only in protists of the groups Kinetoplastea and Diplonemida, such as the genera *Leishmania* and *Trypanosoma* [7]. *Leishmania* FBPase (*Lm*FBPase)-null promastigotes were shown to be internalized by macrophages and to differentiate into amastigotes, but were unable to replicate in the macrophage phagolysosome and failed to generate lesions in mice [8].

Mammalian FBPases (especially pig and human FBPases) are enzymologically and structurally well characterized [9], [10]. The enzymes occur as very similar liver and muscle isoforms and are homotetramers assembled as a pair of dimers. In R-state structures of the liver isoform, the tetramer adopts a planar conformation of the dimers. However, the recently reported R-state structure of muscle FBPase adopts a conformation in which the pairs of dimers are perpendicular to each other [10]. AMP is an allosteric inhibitor of both liver and muscle forms that binds at a site 30 Å from the active site [11]. Binding of AMP induces a conformational change of the tetramer from either a planar or a perpendicular R-state to a twisted T-state [10], [12], [13]. Despite sharing a conserved sequence (77% identity), the muscle FBPase is 100-fold more sensitive to AMP inhibition than its liver counterpart and is inhibited by calcium ions [14]. The allosteric AMP binding site in human liver FBPase is an antidiabetic drug target, and structure-based design approaches have been used to develop small-molecule inhibitors which are currently being evaluated in the clinic [15], [16], [17], [18].

The R-state and T-state structures presented here for *Lm*FBPase are similar to the canonical structures of human liver FBPase, with the R-state tetramer adopting a relatively planar conformation and the inhibited AMP-bound T-state adopting a twisted conformation. However, the biochemical and structural data on *Lm*FBPase show differences in the allosteric mechanism of AMP inhibition compared with the human enzymes. Given the importance of *Lm*FBPase to proliferation of the human pathogenic form of the parasite and formation of lesions, these structural and mechanistic differences suggest that the *Lm*FBPase allosteric AMP-binding pocket could provide a potential new species-specific target for anti-leishmaniasis drugs.

## Results and Discussion

### Sequence alignment of FBPases from different organisms

**Fig. 1:**
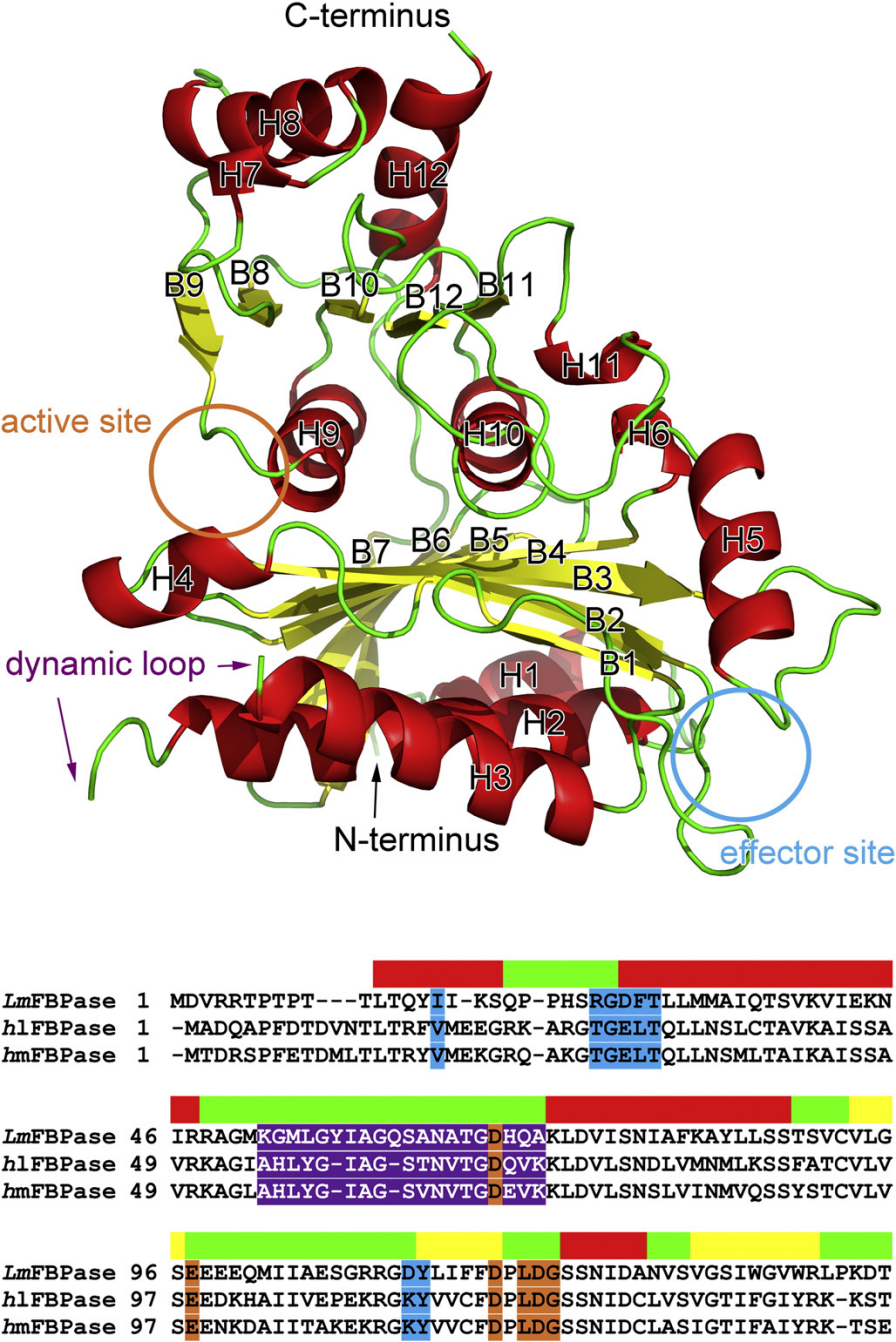
Overall structure of a monomer of *Lm*FBPase. The helices, strands, and loop regions are labeled and are shown in red, yellow, and green cartoon ribbons, respectively. These secondary structure elements are also indicated in the alignment by the same colors. The positions of both ends of the “dynamic loop” (residues 52–71) are shown with purple arrows in the figure, whereas its sequence is highlighted by purple background in the alignment. The position of the active site is shown with an orange circle in the structure, whereas its residue codes are highlighted by orange background in the alignment. Similarly, the effector site is shown in cyan. Sequence alignment between *Lm*FBPase and human was performed with MUSCLE [19]. The terminal residues 1–7 and 338–350 as well as the C-terminal His-tag were not built into the structure models due to the lack of electron density.

Sequence alignment between *Lm*FBPase and mammalian FBPases (Fig. 1, Fig. S1, and Table S1) shows that the enzymes are moderately conserved, despite the long evolutionary divergence between trypanosomatids and mammals. Moreover, *Lm*FBPase is essentially equidistant from human liver FBPase (*h*lFBPase) and human muscle FBPase (*h*mFBPase), with 44% and 41% overall sequence identity, respectively. The active sites from *Lm*FBPase and mammalian FBPases are very well conserved, with 13 out of 14 residues being identical, except for the replacement of Tyr221 by Asn in *Lm*FBPase and *Escherichia coli* FBPase (all residues are numbered according to *Lm*FBPase in this paper, unless stated otherwise). The dynamic loop (residues 52–71), which has been shown to be catalytically important in mammalian FBPases, is in a similar position in *Lm*FBPase but shows functionally important sequence differences and has two insertions (Tyr57 and Gln61) compared with mammalian and bacterial sequences (Fig. S1). The AMP binding sites of *Lm*FBPase and mammalian FBPases are relatively poorly conserved. Intriguingly, the basic residue Lys112, which was predicted to be one of three conserved residues required for the inhibitory effect of AMP [20], is replaced by an acidic residue Asp112.

### Enzymatic characterization of *Lm*FBPase

**Fig. 2:**
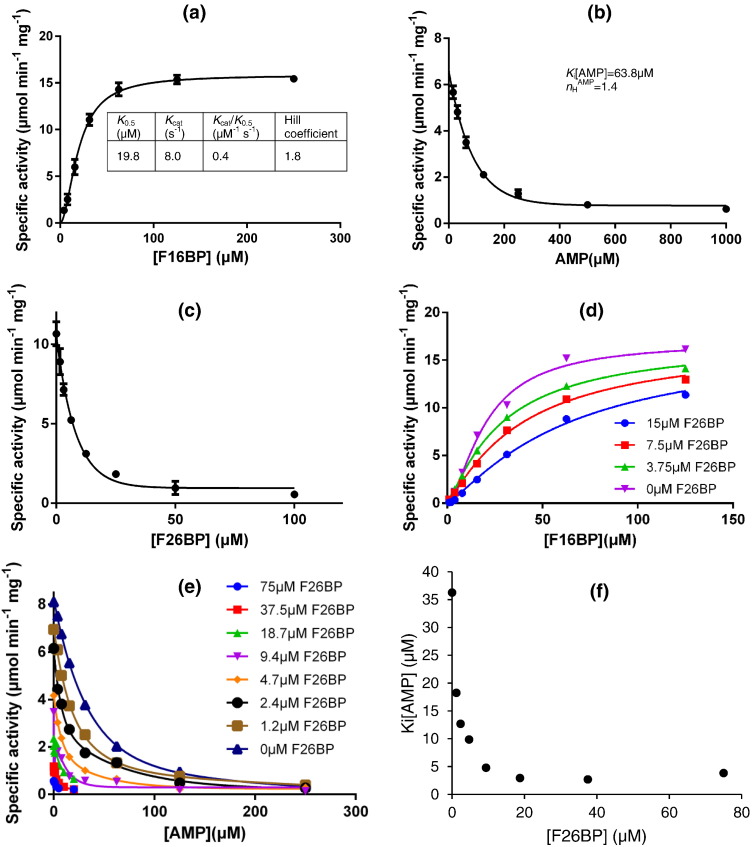
Kinetic characterization of *Lm*FBPase. (a) Determination of the values of kinetic parameters of *Lm*FBPase with its substrate F16BP. (b) The inhibitory effect of AMP on *Lm*FBPase. (c) The inhibitory effect of F26BP on *Lm*FBPase activity. (d) Kinetic characterization of *Lm*FBPase in the presence of different concentrations of F26BP. (e) The effect of AMP titration on the specific activity of *Lm*FBPase in the presence of different concentrations of F26BP. (f) The *K*_i_ of AMP for *Lm*FBPase decreases when higher concentrations of F26BP are present.

A plate-based enzymatic activity assay has been developed for trypanosomatid FBPases by monitoring the formation of NADPH produced via the coupling enzymes phosphoglucose isomerase (PGI) and glucose-6-phosphate dehydrogenase (G6PDH). The activity assays show that the substrate F16BP binds to the enzyme with positive cooperativity (*n*_H_ = 1.8). The *K*_0.5_ value of 19.8 μM (Fig. 2) is similar to that of *E. coli* FBPase isotype I, but considerably larger than those of the mammalian FBPases, which have *K*_m_ values of around 1 to 2 μM (Table 1). The higher *K*_m_ for *Lm*FBPase may be explained by a sequence difference at the active site (Fig. S1), with Tyr221 replaced by Asn221 resulting in the loss of a direct hydrogen bond with the F16BP substrate (discussed later in conjunction with the X-ray results).

**Table 1:** Kinetic parameters for *Leishmania major*, mammalian and bacterial FBPases

	*K*_cat_ (s^− 1^)	*K*_0.5_ (μM)	*K*_cat_/*K*_0.5_ (M^− 1^ s^− 1^)	*n*_H_ [Mg^2 +^]	*K*_a_[Mg^2 +^] (mM)	*K*_i_ [AMP] (μM)	*n*_H_ [AMP]	Reference
*L. major*	8.0	19.8	4.0 × 10^5^	1.8	1.2	63.8	1.4	This study
Human isotype I	20.8	2.1	9.9 × 10^6^	1.8	0.2	4.4	2.1	Rakus *et al*. [11]
Human isotype II	23.4	1.3	1.8 × 10^7^	1.9	0.2	0.1	1.8	Rakus *et al*. [11]
Pig isotype I	20.0	1.2	1.7 × 10^7^	1.7	0.8	0.6	2.2	Iancu *et al*. [21]
*E. coli* isotype I	14.6	15.4	9.5 × 10^5^	ND^a^	0.6	2.7	1.1	Kelley-Loughnane *et al*. [22]

a ND, not determined.

Magnesium is required for catalytic activity of *Lm*FBPase, and a Michaelis–Menten plot of activity *versus* magnesium concentration gives a *K*_m_ value of 1.2 mM (Fig. S2a). Manganese, however, showed an inhibitory effect on *Lm*FBPase activity with a *K*_i_ of 44.3 μM (Fig. S2b). No data for manganese levels in leishmanial promastigotes or amastigotes have been determined; however, measurements on *Trypanosoma brucei* [23] estimated levels of ~ 1 μM manganese in the cytosol. It is unlikely, therefore, that manganese inhibition of *Lm*FBPase is physiologically relevant. Calcium also inhibits *Lm*FBPase activity, with an inhibitor constant similar to that of manganese (Fig. S2b). Both mammalian FBPase isoforms are also inhibited by calcium but with a much stronger inhibition against muscle FBPase (*K*_i_ = 1 μM) than against liver FBPase (*K*_i_ > 1 mM) [14].

### Inhibitory effects of AMP

AMP binds to an effector site about 30 Å distant from the active site and its allosteric inhibition is well characterized in mammalian FBPases [20]. Sequence alignment of mammalian and *Lm*FBPase shows that the residues involved in AMP binding are poorly conserved (Fig. 1), with only three identical out of eight amino acids. Gao *et al*. [20] showed that in mammalian FBPases, AMP inhibition requires serine or threonine as position 31 (position 29 in *Lm*FBPase), lysine or arginine at position 112, and tyrosine at position 113. *Lm*FBPase retains Thr29 and Tyr113, but has an aspartic acid at position 112 instead of a basic residue. The lack of sequence conservation at the effector site explains the weaker binding of AMP to *Lm*FBPase than to mammalian FBPases (*K*_i_ = 63.8 μM compared with 0.1–4 μM). The allosteric inhibitory effect of AMP on *Lm*FBPase is shown in Fig. 2b, and the determined *K*_i_ value of 63.8 μM is in the likely physiological range of cellular AMP concentration in trypanosomatids (estimated to be between 250 and 2000 μM in different growth phases of *T. brucei*) [24]. This suggests that the FBPase activity and thus gluconeogenesis is under AMP regulation in trypanosomatids.

### The synergistic inhibitory effect of F26BP with AMP

Fructose 2,6-bisphosphate (F26BP) binds at the active site, and acts as a competitive inhibitor of both liver and muscle isoforms of mammalian FBPases with inhibition constants of 0.5 and 0.2 μM, respectively [25], [26], [27], [28], [29]. In this study, F26BP also shows an inhibitory effect on *Lm*FBPase, with a *K*_i_ of 1.9 μM (Fig. 2c). A kinetic characterization of *Lm*FBPase in the presence of different F26BP concentrations demonstrated that F26BP reduces the substrate binding affinity without altering the maximum activity (Fig. 2d), confirming that F26BP is a competitive inhibitor of *Lm*FBPase activity.

AMP inhibition of mammalian liver FBPase is enhanced up to 10-fold in the presence of F26BP, in an effect termed AMP/F26BP synergism [25], [26]. Here we show AMP/F26BP has a similar synergistic effect on *Lm*FBPase. A *K*_i_[AMP]-*versus*-[F26BP] graph (Fig. 2f) clearly shows the trend that the AMP inhibitory effect is F26BP dependent. In the presence of F26BP, the *K*_i_ for the AMP falls by up to 13-fold.

### Ligands greatly affect the thermostability of *Lm*FBPase

**Fig. 3:**
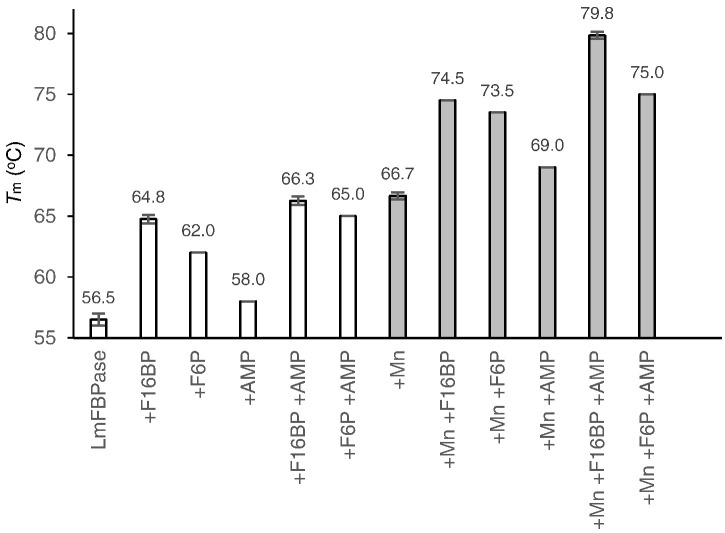
Melting temperatures (*T*_m_) of *Lm*FBPase in the absence and presence of ligands (2 mM) and/or Mn^2 +^ (10 mM). The shaded bars correspond to the presence of Mn^2+^. The thermostability of *Lm*FBPase and the effects of ligands and/or Mn^2+^ were determined with a thermal shift assay (see Materials and Methods for details).

The velocities of *Lm*FBPase unfolding in the absence and presence of different ligands and/or Mn^2+^ as a function of increasing temperature were determined with a thermal denaturation assay (TDA). The results (Fig. 3) show that in the absence of ligands, the melting temperature (defined as the temperature midpoint for the protein unfolding transition [30]) of *Lm*FBPase was 56.5 °C. Thermostability in the presence of the substrate F16BP was increased strikingly for *Lm*FBPase with a Δ*T*_m_ of about 8 °C. In contrast, the inhibitor AMP caused only a modest change in the stability of the protein. Furthermore, the stabilization effect of F16BP was not eliminated by AMP, but the stabilizing effects were additive. This result implies that AMP inhibits the catalytic activity of *Lm*FBPase without eliminating the substrate binding. The catalytic product F6P also showed a stabilization effect on *Lm*FBPase, but not as significant as the substrate F16BP. This result suggests that the 1′-phospho group of F16BP may also play an important role in the stabilization of *Lm*FBPase.

The thermostability of *Lm*FBPase was increased by more than 9 °C in the presence of Mn^2 +^, similar to the increase from the binding of F16BP. The thermostability of *Lm*FBPase plus Mn^2 +^ was also determined in the presence of natural ligands. F16BP in the presence of Mn^2 +^ showed a further stabilization of *Lm*FBPase by increasing the *T*_m_ by about 8 °C, whereas AMP caused only a modest thermostability change. The largest shift in thermostability of more than 20 °C occurred in the presence of Mn^2 +^, F16BP plus AMP. Due to the strong stabilization effect of Mn^2 +^, it was used as a “silver bullet” to crystallize one of the *Lm*FBPase crystal forms.

### Apoenzyme structure of *Lm*FBPase

**Fig. 4:**
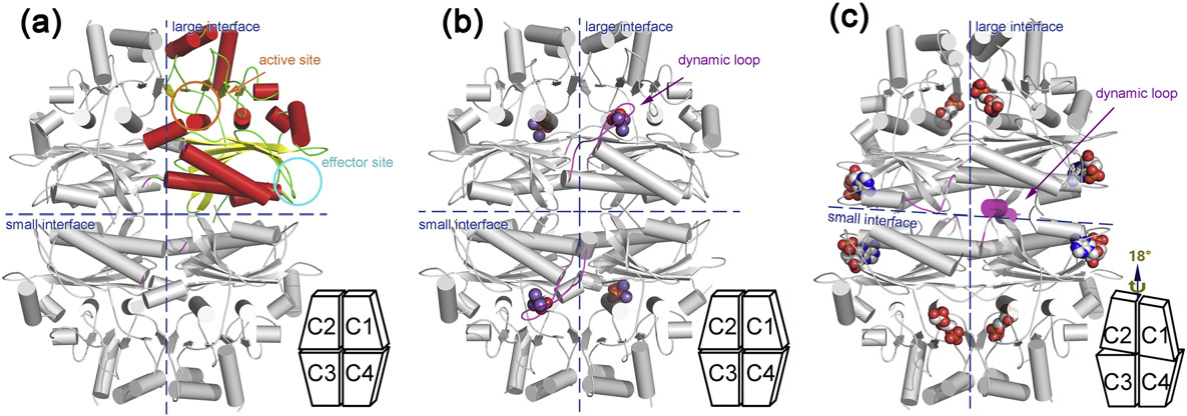
Overall structures of *Lm*FBPase. The large interface [between the Chain 1/Chain 4 (C1C4) dimer and the C2C3 dimer] and small interface (between the C1C2 dimer and the C3C4 dimer) are shown in dashed lines in each homotetramer. Positions of active sites and effector sites are highlighted by arrows and circles in the first figure, where secondary structures are shown in different colors in Chain 1 (helices, β-strands and loop regions in red, yellow, and green, respectively). Bound ligands are shown as spheres. The dynamic loops (residues 52–71) are indicated in purple. Schematic models (adapted from diagrams in Gidh-Jain *et al*. [12]) are shown in the bottom right of each panel. (a) *Lm*FBPase structure in an unligated state. (b) Structure of *Lm*FBPase–phosphate–Mn^2 +^ complex. (c) The structure of *Lm*FBPase–AMP–F6P complex is in an allosterically inhibited conformation.

Three crystal structures of *Lm*FBPase have been determined: an apoenzyme structure that is in an unligated state, a structure bound with its catalytic product, and a structure in its allosterically inhibited form (Fig. 4). All three crystallize in space group *P*2_1_2_1_2_1_ with one tetramer per asymmetric unit. The terminal residues 1–7 and 336–350 as well as the C-terminal His-tag were not built into the structure models due to the lack of interpretable electron density.

The crystal structure of *Lm*FBPase apoenzyme was solved to 2.41-Å resolution using molecular replacement with reference to a monomer of a pig liver FBPase structure (PDB code: 5FBP) [31]. The monomer core structure of *Lm*FBPase has an α/β/α/β/α “club sandwich” topology, with two mixed β-sheets flanked by α-helices on both sides (Figs. 1 and 4a). The active site is located at the end of the B9 strand and adjacent to the N-terminus of the H9 helix. The effector site is located between the termini of helices H1 and H2, and incorporates the seven-residue effector loop (residues 20–26) joining these two helices. The hanging drop in which the apoenzyme structure was crystallized contained F16BP and Mg^2^^+^; however, no ligands are observed in the structure. The topology of the apoenzyme tetramer is planar (Fig. 4a), but is rotated in the presence of the allosteric inhibitor AMP (discussed below).

The homotetramer of *Lm*FBPase is formed from two types of dimer as shown by the schematic models in Fig. 4. The Chain1Chain2 dimer is on the top and the Chain3Chain4 dimer on the bottom, and form the "small interface" (2507 A^2^) between these dimers. Similarly the C2C3 dimer is on the left and the C1C4 dimer on the right, and form the "large interface" (3850 A^2^) between these dimers. The active site is located close to the large interface and comprises residues from adjacent monomers. Small interface interactions mainly involve helices H1, H2, and H3 and strands B6 and B7 (Fig. 1). In the apoenzyme structure, the effector loop “^20^QPPHSRG^26^” between helices H1 and H2 adopts a conformation that strengthens the interactions across the small interface. Movement of this loop is critical for the allosteric inhibition mechanism triggered by AMP binding.

**Fig. 5:**
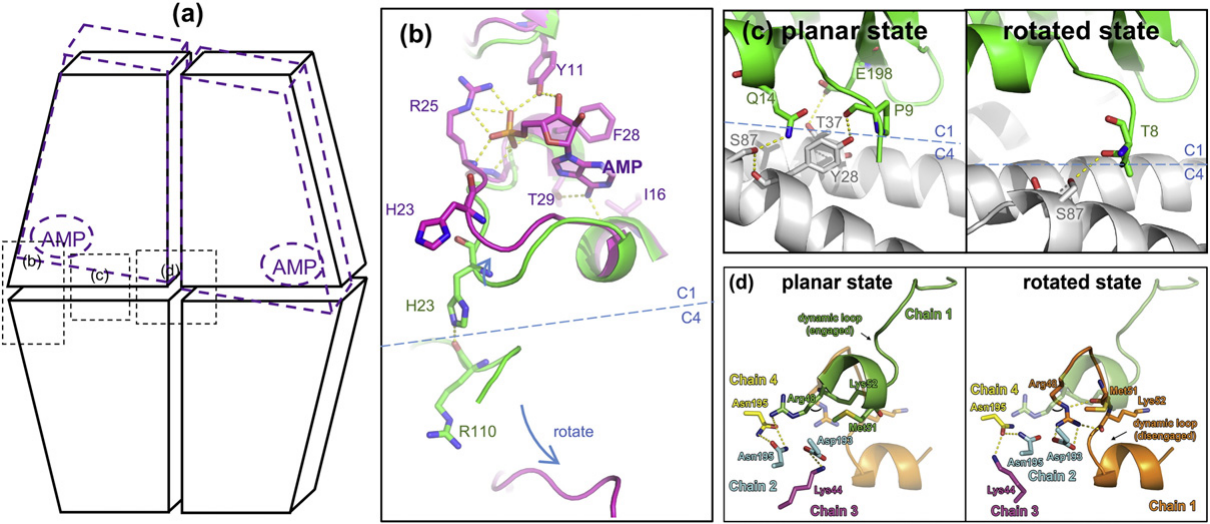
Crystal structures show allosteric effects of AMP on *Lm*FBPase. (a), Schematic representations of the *Lm*FBPase structures. Each trapezoid block represents a chain in this homotetramer (adapted from diagrams in Gidh-Jain *et al*. [12]). Black blocks show the planar topology, while the purple, rotated blocks represent the rotated state. AMP binding sites are indicated with purple ellipses. Positions corresponding to panels b, c, and d are also indicated with black dashed rectangles. (b) Comparison of the AMP binding sites in the planar state structure (green) and the rotated state structure (purple). The planar state and the rotated state structures are superposed on Chain 1 (C1). The blue dashed line shows the short interface. (c) Comparison of the short interfaces of the planar state (left) and rotated state (right) structures. C1 chains are shown in green and purple for the planar and rotated state structures, respectively. C4 chains are shown in gray in each figure. The blue dashed line shows the short interface. (d) Interactions between all four chains in the central region of *Lm*FBPase. (Left) Hydrogen bonds in the central region of the planar state of *Lm*FBPase involve all four chains. These form two independent, but identical symmetry-related hydrogen bond clusters. Chains 1, 2, 3, and 4 are represented in green, cyan, magenta, and yellow, respectively. For the convenience of comparison, the relative position of the dynamic loop of rotated-state *Lm*FBPase is shown in transparent orange. (Right) The same region but in the rotated inactive T-state has a different network of hydrogen bonds because the side chain of Arg48 has switched to a different position upon AMP binding. Chains 1, 2, 3, and 4 are represented in orange, cyan, magenta, and yellow, respectively. For the convenience of comparison, the relative position of the dynamic loop of planar-state *Lm*FBPase is shown in transparent green.

Intriguingly, not only interactions across both the small and the large interfaces, but also interactions between diagonal chains are formed. Two independent, but identical symmetry-related clusters of hydrogen bonds, each lying across the 2-fold symmetry axis of the tetramer, are formed by Asn195 and Arg48 (Fig. 5d). The rearrangement of these eight hydrogen bonds that stabilize the core of the homotetramer in the planar R-state regulates the R to T switching mechanism (Fig. 5d, discussed below).

### Crystal structure of *Lm*FBPase in complex with inorganic phosphate and metal ions

**Fig. 6:**
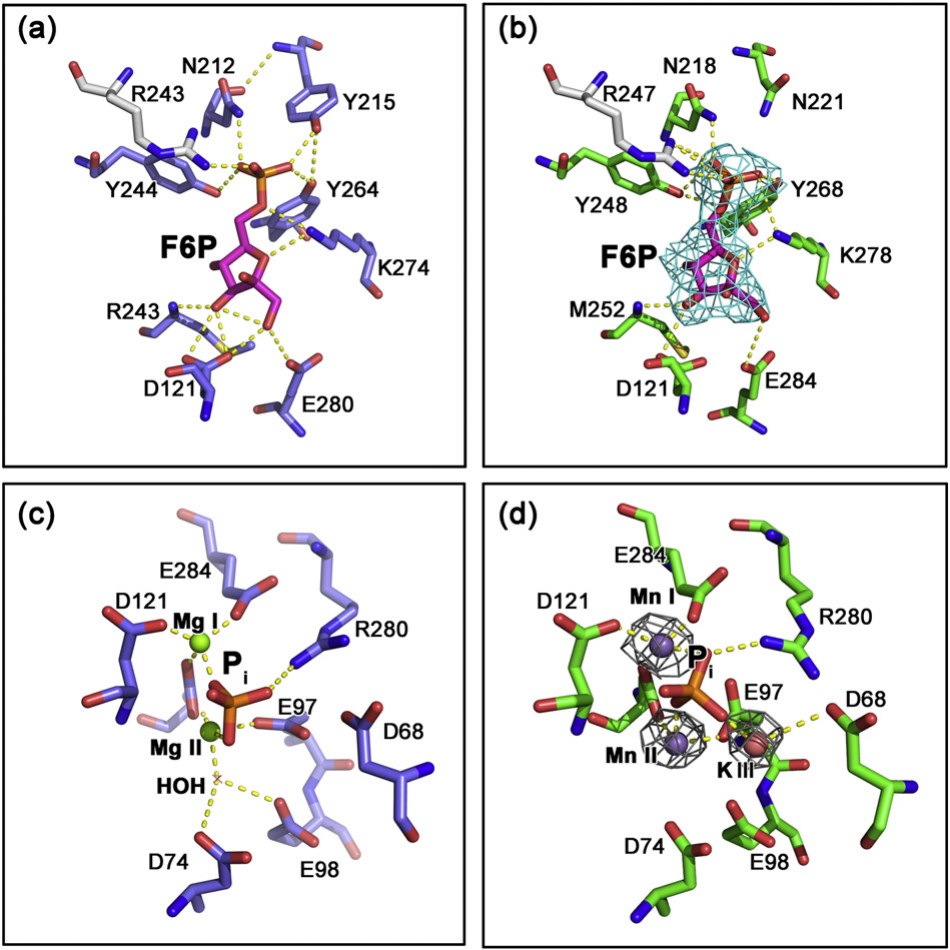
Comparison of the active sites of pig liver FBPase (panels a and c, blue; generated from the work by Choe *et al*. [33]; PDB code: 1EYI] and *L. major* FBPase (panels b and d, green; generated from this study). F6P molecules are shown in magenta. Arginines from adjacent chains are shown in gray. The electron density of F6P in panel B (colored in cyan) is from *F*_o_ − *F*_c_ maps contoured at the 3.0*σ* level. Electron density at the metal sites in panel d (gray mesh) is from 2*F*_o_ − *F*_c_ maps contoured at the 3.5σ level.

Manganese was demonstrated by TDA measurements to be a strong stabilizer of *Lm*FBPase (Fig. 3) and was used as an additive to crystallize *Lm*FBPase. Co-crystallization of *Lm*FBPase with Mn^2 +^ and the substrate F16BP resulted in crystals in which a catalytic product P_i_ and manganese atoms were found in each active site (shown in Fig. 6b, d). The overall conformation is very similar to the apoenzyme structure, with an RMSD value of only 1.0 Å (based on Cα atoms). This structure also adopts a planar topology, which has no significant rotational movement of domains as analyzed by Dyndom [32]. The dynamic loop (residues 52–71) was found to adopt an engaged conformation (Fig. 4b), in contrast to the apoenzyme structure where the dynamic loop was found to be disordered in two of the chains. The dynamic loop is inserted into the active site, showing the involvement of the catalytically critical Asp68 that interacts with a cation at site III which binds to the catalytic product P_i_ in the active site (Fig. 6d).

### Crystal structure of *Lm*FBPase in complex with AMP

The *Lm*FBPase tetramer co-crystallized with the substrate F16BP in the presence of the allosteric inhibitor AMP and manganese adopts a twisted conformation. The catalytic product F6P was found in each of the active sites of the four subunits, and AMP was located in each of the four effector sites at approximately 32 Å away from the active sites (Fig. 4c). Although it did not significantly change the monomer conformation in comparison with the apoenzyme structure (RMSD of 1.0 Å based on Cα atoms), the overall tetramer structure had an RMSD of 3.8 Å (Fig. S3). The difference was a result of AMP binding which induced an 18° rotation, shearing the small interface and rotating the C1–C2 dimer relative to the C3–C4 dimer to yield a “rotated structure”.

As a result of this rotation, the dynamic loop (residues 52–71) is pulled out from the active site in a canonical “disengaged conformation” [33], which is stabilized by the diagonal subunit. Removal of key residues (especially Asp68) of the dynamic loop from the active site deactivates *Lm*FBPase. The “disengaged-conformation” (in which most of the loop is disordered in the AMP-bound structure) is prevented from adopting an active conformation by the strong hydrogen bonded network around Arg48 which hydrogen bonds to the dynamic loop (residue 52) (Fig. 5d) and pointing the remainder of the loop out into solvent. The conformations of the dynamic loop identified in the three different crystal structures show the key catalytic role the loop plays in the allosteric FBPase mechanism: the hydrogen bond interactions between Arg48 and the backbone carbonyl oxygen atoms of Met51 and Lys52 tie down the disordered loop in an inactive conformation (Fig. 5d). There is also a strong salt bridge between Arg48 and Asp193 from Chain 2 across the large interface. In the R-state, the Arg48 side chain rotates 180° round the Χ_11_ to release the disordered loop and help lock the tetramer in a planar conformation by forming a hydrogen bond to Asn195 (Chain 4) across the short interface (Fig. 5d).

As found in both mammalian and *Leishmania* FBPases, the formation of the complete active site is facilitated by Arg247 from the adjacent subunit across the large interface, which forms an ionic interaction with the 6′-phospho group of the catalytic product F6P (Fig. 6a, b). This interaction also demonstrates that the tetrameric form of *Lm*FBPase is necessary for catalysis. The sequence alignment (Fig. 1) shows that the active sites among *Leishmania* and mammalian FBPases are conserved, except for a one-residue difference—the tyrosine that is responsible for binding to the 6′-phospho group of the catalytic substrate/product in mammalian FBPases is replaced by Asn221 in *Lm*FBPase. The shorter side chain of Asn221 may explain the weaker substrate binding affinity of *Lm*FBPase compared with mammalian FBPases affinity as Asn221 cannot make a direct hydrogen bond with the F16BP substrate (Fig. 6). Similar catalytic properties of *E. coli* FBPase, which has a corresponding asparagine in its active site [22], are consistent with this explanation.

### Conformational variability of the FBPase active site

The three *Lm*FBPase structures are compared with corresponding mammalian FBPase structures in Table 2. Co-crystallization of *Lm*FBPase with F16BP, magnesium, and potassium resulted in an apoenzyme structure containing no substrate, product, or metals and no observable electron density for the dynamic loop (for two of the chains). This apoenzyme form of *Lm*FBPase is similar to the apoenzyme (R-state) planar conformer of pig kidney FBPase (PDB code: 2FBP), where the dynamic loop is also completely disordered [28].

**Table 2:** Comparisons of crystal structures of *L. major* and mammalian FBPases

Structure name	*Lm*FBPase apoenzyme	*p*lFBPase apoenzyme	*Lm*FBPase/Mn/P_i_	*p*lFBPase/F6P/P_i_/Mg	*Lm*FBPase/F6P/AMP	*p*lFBPase/F6P/P_i_/AMP/Mg
PDB code	5OEZ	2FBP	5OEY	1EYI	5OFU	1EYJ
Species/isoform	*L. major*	Pig liver	*L. major*	Pig liver	*L. major*	Pig liver
Space group	*P*2_1_2_1_2_1_	*P*3_2_21	P2_1_2_1_2_1_	*I*222	*P*2_1_2_1_2_1_	*P*2_1_2_1_2
Topology	Planar	Planar	Planar	Planar	Rotated	Rotated
Ligand(s) in crystallization drop	MgCl_2_, KCl, F16BP	None	MgCl_2_, MnCl_2_, KCl, F16BP	MgCl_2_, F6P, KP_i_	MgCl_2_, MnCl_2_, KCl, F16BP, AMP	MgCl_2_, F6P, KP_i_, AMP
Ligand(s) in structure	None	None	P_i_; metal site 1: Mn; metal site 2: Mn; metal site 3: unknown blob	F6P; P_i_; metal site 1: Mg; metal site 2: Mg; metal site 3: unknown blob	F6P; AMP	F6P; P_i_; metal site 1: Mg; AMP
Dynamic loop	Disordered	Disordered	Engaged	Engaged	Disengaged	Disengaged
Reference	This study	Ke *et al*. [27]	This study	Choe *et al*. [33]	This study	Choe *et al*. [33]

The dynamic loop of *Lm*FBPase was found to be stabilized in an engaged conformation in the presence of inorganic phosphate and manganese (Fig. 4b). This corresponds to the observation that manganese dramatically increases the thermostability of *Lm*FBPase (Fig. 3). The catalytic product P_i_ is found in the active site, surrounded by two (or possibly three) manganese atoms (Fig. 6d). In mammalian FBPase, three divalent metal ions are required for hydrolysis of F16BP [33]. Indeed, three electron density peaks were found in each active site of this *Lm*FBPase/Mn/P_i_ structure; anomalous difference maps calculated from data collected at 1.89 Å (the absorption edge for Mn) showed clear peaks consistent with manganese ions at metal sites I and II. The electron density at site III is only slightly less than the electron density for the two manganese ions; however, there is no observable anomalous signal for site III, making potassium the more likely candidate (Fig. 6d). This assignment is also supported by the fact that potassium is required for enzyme activity and is present at a concentration 100 mM in the enzyme assay conditions and was present in the crystallization solution at 50 mM.

In the pig liver FBPase structure, an important water molecule was found as a bridge between Asp74 and metal II (Fig. 6c). This water molecule is presumed to act as a catalytic base in the abstraction of a proton from the second coordinated water molecule (coordinated with Glu98) [33]. Similar coordination geometries around Mn-I and Mn-II are found in the *Lm*FBPase crystal structures with Mn–O distances close to 2.2 Å [34]. Although Mn^2 +^ was originally shown to act as a cofactor for mammalian FBPase activity [35], here we show that at higher concentrations Mn^2 +^ is an inhibitor of *Lm*FBPase with a *K*_i_ ~ 45 μM (Fig. S2b).

In mammalian FBPases, a divalent metal ion is retained at the metal site I in both R- and T-state structures and remains coordinated with F6P and P_i_, as well as three acidic amino acids Asp118, Asp121, and Glu280. In contrast, in the T-state structure of *Lm*FBPase/F6P/AMP, no metal atoms are found in the active site. This rotated structure with a twist of 18° adopts a similar conformation to the rotated conformation of pig liver FBPase (RMSD value of 0.85 Å), which has a twist of 15°.

### Allosteric mechanism of *Lm*FBPase

In mammalian FBPase structures, two canonical structural states have been identified. The active state structure (R-state) showed a planar tetramer conformation, whereas a 15° rotation of the C1–C2 dimer relative to the C3–C4 dimer was demonstrated to take place in order to form the inactive state structure (T-state) [12], [31], [33], [36]. The structural superposition between tetramer coordinates confirmed that the apoenzyme structure of *Lm*FBPase is more like the active state (RMSD value of 1.1 Å) than the inactive state (RMSD value of 3.7 Å) of pig liver FBPase.

In this study, both the apoenzyme structure of *Lm*FBPase and the complex structure with P_i_ and manganese adopt planar conformations, whereas the binding of AMP at the effector site induces an 18° rotation along the small interface of the homotetramer (animation shown in Supplementary Movie). As shown in Fig. 5a, the conformational change from the planar conformation (represented by black solid trapezoid blocks) to the rotated conformation (represented by purple dashed blocks) mainly takes place along the short interface. In the planar structure (Fig. 5b, green), with no AMP bound, the position of the effector loop region between helices H1 and H2 is toward the C1–C4 short interface. Within this loop, His23 makes a hydrogen bond with the main-chain oxygen of Arg110 across the short interface and helps lock the interface interaction in the planar R-state. When AMP binds to the adjacent AMP binding pocket (shown in magenta), the effector loop makes a large conformational change and loop residue Thr29 forms a hydrogen bond with the adenine ring, while Arg25 forms a salt bridge with the phospho group. This effector loop movement breaks the cross-interface interactions pulling the “His23-lock” away from the interface. The unlocked chain (C4) becomes free to rotate. This “unlock and rotate” mechanism appears unique to the *Lm*FBPase structure as there is little conservation of amino acid sequence in the corresponding effector loops (residues 20–26) of the mammalian enzymes. In both liver and muscle FBPase structures, helix H1 is extended by one turn compared with *Lm*FBPase in which Pro21 acts as a helix breaker and serves to shorten the H1 helix, thereby extending the effector loop (Fig. S4). This proline is conserved in both *T. brucei* and *Trypanosoma cruzi* sequences.

In the planar structures (Fig. 5c, green), the small interface is stabilized by hydrogen bonds across the small interface (including Gln14–Ser87, Glu198–Thr37, Pro9–Tyr28). Upon AMP binding and dimer rotation, these interactions are disrupted and a new hydrogen bond Thr8–Ser87 is formed. These hydrogen bond changes across the small interface are also coupled with the Arg48 conformational switch (described above and in Fig. 5d), which ties the dynamic loop in the “disengaged conformation” (Fig. 5d).

The residue corresponding to Arg48 in *Lm*FBPase is conserved in mammals and many other organisms (e.g., *E. coli* and *Schizosaccharomyces pombe*, shown in Fig. S5). Interestingly, in representatives of all genera of the Kinetoplastea analyzed, whether belonging to the trypanosomatid or bodonid families, Arg is found, except in *Trypanosoma* (Fig. S5). In both African and American trypanosomes, either Ser or Thr is found at the position corresponding to *Lm*Arg48, suggesting that a common ancestral species of the genus *Trypanosoma* has lost Arg. Preliminary kinetic analysis of *T. brucei* FBPase has shown that the enzyme is much less sensitive to the AMP inhibitor, with only 50% enzyme activity inhibition found at 0.8 mM AMP, at optimal substrate concentration. The Arg mutation would explain this loss of sensitivity as neither Ser nor Thr would be able to form a salt bridge across the large interface and simultaneously lock down the dynamic loop in the disengaged conformation.

### Structurally distinct effector site offers a drug target against *Lm*FBPase

**Fig. 7:**
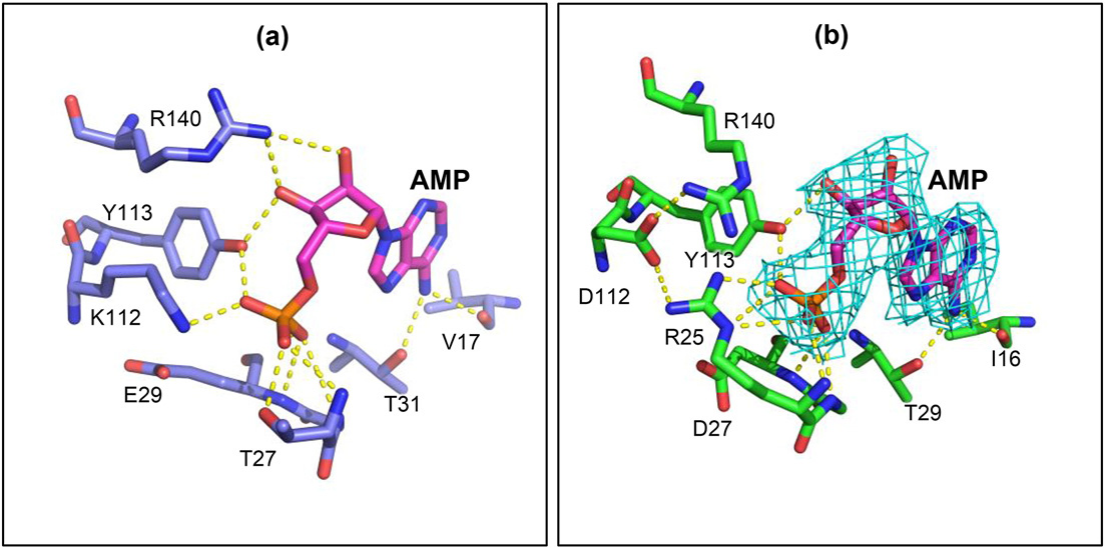
Comparison of AMP binding sites. (a) Conformation of the AMP binding site in human muscle FBPase [37] (blue; PDB code: 3IFA). (b) Conformation of the AMP binding site in *Lm*FBPase (green). The electron density shown is from *F*_o_ − *F*_c_ maps contoured at the 3.0σ level.

Despite similarities in allosteric mechanisms of *Leishmania* and mammalian FBPases, the AMP allosteric binding sites show important differences (Figs. 7 and S4). Zarzycki *et al*. [37] solved a human FBPase crystal structure in complex with AMP (Fig. 7a) and showed that seven residues are responsible for the binding of AMP. The notably different AMP binding site in *Lm*FBPase has six interacting residues (Fig. 7b), of which only two (Tyr113 and Thr29) are conserved suggesting their potential evolutionary importance. A sequence alignment of 307 FBPases from different organisms performed by Gao *et al*. showed the conservation of these two residues.

The main difference in the AMP effector site is the switch from Lys112 and Thr27 in *h*FBPase to Asp112 and Arg25 in *Lm*FBPase (Fig. 7). This results is an important change in the conformation of Arg140 which interacts with the two hydroxyl groups of AMP in *h*FBPase but forms a salt bridge with Asp112 in *Lm*FBPase and no longer binds with AMP, thereby losing two direct hydrogen bonds with AMP, which is consistent with its weaker binding (Table 1).

An FBPase null cell line of *Leishmania major* showed a loss of ability to replicate in the macrophage phagolysosome and an inability to cause lesions in infected mice [8]. These observations make *Lm*FBPase an interesting drug target for diseases caused by *Leishmania* species.

Drugs targeting the AMP site of human FBPases have been extensively studied and designed for the treatment of type 2 diabetes. For instance, benzoxazole benzenesulfonamides [38], imidazole analogues [39], and some tricyclic compounds [40] have been demonstrated to bind to the effector site of human liver FBPases with affinities at the nanomolar level. Among these human FBPase inhibitors, some of them (e.g., CS-917 under clinical phase 2b trials) have gained particular success [41].

In this study, we showed that the *Leishmania* FBPase effector site is structurally distinct from the host FBPase isoforms and that the allosteric inhibition mechanisms are also different. The structural differences at the effector site suggest that a species-specific inhibitor could be developed. Evaluation of the “druggability” of the effector pocket using a number of prediction programs (Fig. S6) suggests that the effector site of *Lm*FBPase is at least as druggable as the proven human target of liver FBPase.

## Materials and Methods

### Gene cloning, overexpression, and protein purification

The gene encoding *L. major* FBPase was amplified by PCR using *L. major* (MHOM/SU/73/5-ASKH) genomic DNA. The amplified fragments were ligated into a pET24a vector (Novagen), which contains the coding sequence for a C-terminal His_6_ tag. The overexpression plasmid was used to transform *E. coli* BL21(DE3) Gold strain (Novagen). Single colonies were picked from LB kanamycin (50 μg/ml) plates and used to inoculate 50 ml of LB medium (containing kanamycin). Cultures were grown overnight at 37 °C and 250 rpm. Aliquots (10 ml) were used to inoculate 500 ml of LB medium (containing kanamycin) in 2-L Erlenmeyer flasks. The cell culture was grown at 37 °C and 250 rpm to an OD_600_ of 1.0, at which time *Lm*FBPase expression was induced by the addition of isopropyl-β-d-thiogalactopyranoside (final concentration 1 mM). The culture was maintained (at 30 °C and 120 rpm) for an additional 24 h. The cells were then harvested, followed by lysing with a constant cell disruption system in lysis buffer [50 mM triethanolamine (TEA), 300 mM NaCl, 20 mM imidazole, and 1 tablet EDTA-free protease inhibitors (Roche), pH 8.0] at 25 MPa. The lysate was centrifuged at 6 °C for 45 min at 60,000*g*, and the supernatant was filtered through a 0.2-μm syringe filter. The C-terminal-His_6_-*Lm*FBPase was purified from lysates using immobilized metal ion affinity chromatography on a nickel affinity resin (GE Healthcare Co., Ltd.), followed by a size-exclusion step with a HiPrep 16/60 S200 column (GE Healthcare Co., Ltd.). Purified protein was concentrated to 5 mg/ml using a centrifugal membrane concentrator (Sartorius Co., Ltd.), and then frozen in liquid nitrogen and stored at − 80 °C.

### Enzyme activity assays

PGI and G6PDH were used as coupling enzymes in assays for *Lm*FBPase. For the determination of its specific activity with the substrate F16BP, reduction of NADP^+^ to NADPH was monitored by absorbance at 340 nm. For the determination of kinetic parameters of *Lm*FBPase, enzyme activity assays were performed as follows. Fifty microliters of different concentrations of F16BP (from 0 to 500 μM) in assay buffer [50 mM TEA, 100 mM KCl, 10 mM MgCl_2_, 5% glycerol (pH 8.0)] were added into each well in a 96-well microtiter plate. Fifty microliters of solution containing 4.6 μg/ml of *Lm*FBPase, 1.4 U/ml of PGI, 0.8 U/ml of G6PDH, and 0.5 mM of NADP^+^ in the same assay buffer were added to each well to start the reaction. The increase in absorbance at 340 nm (the absorbance of NADPH) was measured for 5 min at 37 °C using a plate reader. The kinetic parameters (*V*_max_, *K*_0.5(F16BP)_, and *n*_H_) were determined by curve fitting to the data with the equation below.

$$V=\frac{V_{\max}\times {\left[F16\mathrm{BP}\right]}^{n_{H}}}{{K_{0.5}}^{n_{H}}+{\left[F16\mathrm{BP}\right]}^{n_{H}}}$$

For the determination of the effects of AMP and manganese on *Lm*FBPase activity, 50 μl of different concentrations of AMP or MnCl_2_ (from 0 to 2 mM) in assay buffer in the presence of a sub-saturating concentration of F16BP (40 μM) was added to each well. After 50 μl of mixed solution containing 4.6 μg/ml of *Lm*FBPase, 1.4 U/ml of PGI, 0.8 U/ml of G6PDH, and 0.5 mM of NADP^+^ in the same assay buffer were added to each well to start the reaction, the enzyme activities were assayed at 37 °C. The activation effects of magnesium were also determined using similar methods but at a saturating concentration (250 μM) of F16BP.

### TDA

A fluorescence-based TDA was performed to characterize the thermal stability of *Lm*FBPase with a Bio-Rad iQ5 rtPCR Thermocycler [42]. Each sample contained 0.1 mg/ml of the enzyme, 5 × SYPRO orange (original 5000 × stock from Life Technologies Co., Ltd.), and 2 mM of each ligand (and/or 10 mM of MnCl_2_) in 50 μl of 1 × buffer [10 mM MgCl_2_, 100 mM KCl, 50 mM TEA, 10% glycerol (pH 7.2)]. The 96-well plate containing the samples was heated at rate of 1.0 °C/min from 20 to 80 °C, and the fluorescence intensity was measured with excitation/emission wavelengths of 490 and 580 nm, respectively. Melting temperature (*T*_m_) values were calculated by the software CFX Manager. Samples were tested in duplicate.

### Protein crystallization and data collection

Single crystals of *Lm*FBPase were obtained by vapor diffusion using the hanging-drop technique. (1) The crystals of the apoenzyme structure were formed by mixing 1.5 μl of *Lm*FBPase (3.8 mg/ml of enzyme in 20 mM TEA, 5 mM MgCl_2_, 50 mM KCl, 10% glycerol, 1 mM F16BP, pH 7.0 solution) with 1.5 μl reservoir solution [20% w/v PEG 3350 (0.05 M citric acid, 0.05 M Bis–Tris propane, pH 5.0)] and “silver bullet 85” [43] in crystal growth kit HR2-096 from Hampton Research Co., Ltd. [19 amino acids excluding cysteine in 0.02 M sodium Hepes (pH 6.8)]. The crystals were grown at 17 °C. Plate-like crystals appeared after 3 days. (2) The crystals of *Lm*FBPase/Mn^2 +^/P_i_ were crystallized by mixing 1.5 μl of *Lm*FBPase [4.0 mg/ml of enzyme in 20 mM TEA, 5 mM MgCl_2_, 50 mM KCl, 10% glycerol, 1 mM F16BP, and 10 mM MnCl_2_, (pH 7.0)] with 1.5 μl reservoir solution [20% w/v PEG 3350 (0.05 M citric acid, 0.05 M Bis–Tris propane, pH 6.0)]. Crystals were grown in 3 weeks at 17 °C. Twenty percent of PEG 400 plus 10% glycerol were used as cryoprotectants. (3) For the crystals of *Lm*FBPase/F6P/AMP, 1.5 μl of *Lm*FBPase [3.4 mg/ml of enzyme in 20 mM TEA, 5 mM MgCl_2_, 50 mM KCl, 10% glycerol, 1 mM F16BP, 10 mM MnCl_2_ and 2 mM AMP (pH 7.2)] was mixed with 1.5 μl reservoir solution [19% w/v PEG 3350 (0.05 M citric acid, 0.05 M Bis–Tris propane, pH 6.0)]. Crystals were grown for 4 weeks at 4 °C. The X-ray data sets were collected on Beamline I02 (for the apoenzyme structure), Beamline I04 (for the structure of *Lm*FBPase/Mn^2 +^/P_i_), and Beamline I03 (for the structure of *Lm*FBPase/F6P/AMP) at the Diamond synchrotron radiation facility in Oxfordshire, United Kingdom. Each data set was obtained from a single-crystal flash-frozen in liquid nitrogen.

### Structure determination

The data of the apoenzyme structure were processed with xia2 [44]. The structure was solved with molecular replacement against pig liver FBPase (PDB code: 5FBP) [31] using the program PHASER [45] and the sequence of *Lm*FBPase in Autobuild [46]. The initial models were subjected to a cycle of rigid-body refinement followed by several cycles of restrained refinement using the program REFMAC [47]. The models were then manually adjusted using COOT [48], followed by several cycles of restrained refinement (without NCS restraints) using REFMAC or PHENIX [49]. Water molecules were manually added to the structure using COOT. After additional cycles of restrained refinement and manual adjustments to side chains and water molecules, the overall quality of the map improved. Missing loops were then built up manually using COOT followed by cycles of translation libration screw (TLS) refinement [50] and restrained refinement in REFMAC and COOT adjustments. TLS groups were generated according to the domain region of *Lm*FBPase for each chain using TLS Motion Determination. The first seven TLS groups were used for each structure.

The data of *Lm*FBPase with bound manganese and phosphate ions were processed with MOSFLM [51] and scaled with SCALA [52]. This structure was solved using the coordinates of one monomer from the apoenzyme structure. Briefly, all four subunits were found using the program PHASER, and this initial model was then modified with cycles of manual building with COOT and restrained refinement as described above with the apoenzyme structure. When appropriate, relevant ligand molecules were added where clear unbiased *F*_o_ − *F*_c_ electron density was observed. Water molecules were added to the model using COOT, and after several rounds of restrained refinement, the *R*/*R*_free_ values converged.

The data of *Lm*FBPase with bound F6P and AMP were processed with xia2 [44]. This structure was solved using the coordinates of one monomer from the apoenzyme structure. Like the two solved structures above, four subunits were found in each asymmetric unit. Molecular replacement and structure refinement were performed with PHASER, REFMAC, and COOT. Data collection and refinement statistics are summarized in Table 3. Pair-rotation angles were measured with the Cα atoms of Ala128 and Asp144 between the planar and rotated structures.

**Table 3:** Crystallographic data collection and model refinement statistics.

	*Lm*FBPase apo-structure	*Lm*FBPase + Mn^2 +^+P_i_	*Lm*FBPase + F6P + AMP
PDB code	5OEZ	5OEY	5OFU
Wavelength	0.9795	0.9795	0.9700
Resolution^a^ (Å)	58.77–2.41 (2.496–2.41)	55.14–2.8 (2.9–2.8)	70.25–2.62 (2.714–2.62)
Space group	*P*2_1_2_1_2_1_	*P*2_1_2_1_2_1_	*P*2_1_2_1_2_1_
Unit cell *a*,*b*,*c* (Å)	55.26, 162.56, 170.13	92.01, 104.15, 137.74	90.59 109.25 140.51
Total reflections	290,741 (22,595)	457,591 (67,605)	232,518 (16,569)
Unique reflections	59,755 (5885)	33,215 (3280)	42,513 (4158)
Multiplicity	4.9 (5.2)	13.7 (14.1)	5.5 (5.4)
Completeness (%)	99.4 (99.8)	100 (100)	99.9 (99.6)
Mean *I*/sigma(*I*)	9.2 (2.9)	12.3 (3.5)	12.4 (2.1)
Wilson *B*-factor	50.2	26.5	45.3
*R*_merge_^b^ (%)	9.4 (86.1)	5.4 (74.1)	10.2 (77.3)
*R*_work_^c^	0.187	0.198	0.188
*R*_free_^d^	0.225	0.244	0.216
Number of non-hydrogen atoms	9880	10,120	10,279
Macromolecules	9755 (4 × macromolecules)	9925 (4 × macromolecules)	10,007 (4 × macromolecules)
Waters	125	141	114
Ligands	none	54 (4 × PO_4_, 12 × Mn^2 +^, 2 × Cit)	158 (4 × F6P, 4 × AMP, 2 × Cl)
Protein residues	1244	1287	1282
RMS (bonds, Å)	0.01	0.01	0.009
RMS (angles, °)	1.416	1.4	1.416
Ramachandran favored (%)	98	97	97
Ramachandran allowed (%)	2	3	3
Ramachandran outliers (%)	0	0.1	0
Rotamer outliers (%)	4	5	3
Clashscore	3	4	2
Average *B*-factor	66.10	33.51	56.20
Macromolecules	66.27	33.67	57.46
Ligands	N/A	50.00	49.00
Solvent	52.28	15.25	42.36

a Values in parentheses are for the highest resolution shell.

b *R*_merge_ = Ʃ_*hkl*_ |* I* − <* I* >|/Ʃ_*hkl*_*I*.

c *R*_work_ = Ʃ |* F*_obs_ − *F*_calc_ |/Ʃ |* F*_obs_ |, where *F*_obs_ and *F*_calc_ are the observed and the calculated structure factors, respectively.

d *R*_free_ is calculated using 5% of total reflections randomly chosen and excluded from the refinement.

The molecular interface area is calculated as the difference in total accessible surface areas of isolated and interfacing structures divided by two (calculated with the program PDBePISA [53]).

The following are the supplementary data related to this article.

Supplementary material Image 2

Supplementary movie Animation of structural transitions between T- and R-states of *Leishmania* Fructose-1,6-Bisphosphate. Supplementary movie
