# Supplementary material for: Structures of Leishmania Fructose-1,6-Bisphosphatase Reveal Species-Specific Differences in the Mechanism of Allosteric Inhibition
Source: J Mol Biol. 2017 Oct 13;429(20):3075–89. doi: 10.1016/j.jmb.2017.08.010 (PMC5639204; doi:10.1016/j.jmb.2017.08.010)
Supplement: Supplementary material [file mmc1.docx]

**Supplementary Data**

**Title:** Structures of *Leishmania* fructose-1,6-bisphosphatase reveal species-specific differences in the mechanism of allosteric inhibition

**Authors:** Meng Yuan, Montserrat G. Vásquez-Valdivieso, Iain McNae, Paul A. M. Michels, Linda A. Fothergill-Gilmore, Malcolm D. Walkinshaw

**Affiliation:** Centre for Translational and Chemical Biology, School of Biological Sciences, University of Edinburgh, Michael Swann Building, Max Born Crescent, Edinburgh EH9 3BF, U.K.

**Abstract:** The gluconeogenic enzyme fructose-1,6-bisphosphatase has been proposed as a potential drug target against *Leishmania* parasites that cause up to 20,000 – 30,000 deaths annually. A comparison of three crystal structures of *L. major* fructose-1,6-bisphosphatase (*Lm*FBPase) along with enzyme kinetic data show how AMP acts as an allosteric inhibitor and provides insight into its metal-dependent reaction mechanism. The crystal structure of the apoenzyme form of *Lm*FBPase is a homotetramer in which the dimer of dimers adopts a planar conformation with disordered ‘dynamic loops’. The structure of *Lm*FBPase, complexed with manganese and its catalytic product phosphate shows the dynamic loops locked into the active sites. A third crystal structure of *Lm*FBPase complexed with its allosteric inhibitor AMP shows an inactive form of the tetramer, in which the dimer pairs are rotated by 18° relative to each other. The three structures suggest an allosteric mechanism in which AMP binding triggers a rearrangement of hydrogen-bonds across the large and small interfaces. Retraction of the ‘effector loop’ required for AMP binding releases the side chain of His23 from the dimer-dimer interface. This is coupled with a flip of the side chain of Arg48 which ties down the key catalytic dynamic loop in a disengaged conformation and also locks the tetramer in an inactive rotated T-state. The structure of the effector site of *Lm*FBPase shows different structural features compared with human FBPases, thereby offering a potential and species-specific drug target.

|  |  |  | **/\/\/\/\/========\/\/\/\/\/\/\/\/\/\** |
| --- | --- | --- | --- |
|  | ***Lm*FBPase** | **1** | **MDVRRTPTPT---TLTQYII-KSQP-PHSRGDFTLLMMAIQTSVKVIEKN** |
|  | ***h*lFBPase** | **1** | **-MADQAPFDTDVNTLTRFVMEEGRK-ARGTGELTQLLNSLCTAVKAISSA** |
|  | ***h*mFBPase** | **1** | **-MTDRSPFETDMLTLTRYVMEKGRQ-AKGTGELTQLLNSMLTAIKAISSA** |
|  | ***p*lFBPase** | **1** | **-MTDQAAFDTNIVTLTRFVMEEGRK-ARGTGEMTQLLNSLCTAVKAISTA** |
|  | ***ec*FBPase** | **1** | **-----------MKTLGEFIVEKQHEFSHATGELTALLSAIKLGAKIIHRD** |
|  |  |  | **** .:::: . :. *::* *: :: . * *** |
|  |  |  |  |
|  |  |  | **/\========================/\/\/\/\/\/\///\/====///** |
|  | ***Lm*FBPase** | **46** | **IRRAGMKGMLGYIAGQSANATGDHQAKLDVISNIAFKAYLLSSTSVCVLG** |
|  | ***h*lFBPase** | **49** | **VRKAGIAHLYG-IAG-STNVTGDQVKKLDVLSNDLVMNMLKSSFATCVLV** |
|  | ***h*mFBPase** | **49** | **VRKAGLAHLYG-IAG-SVNVTGDEVKKLDVLSNSLVINMVQSSYSTCVLV** |
|  | ***p*lFBPase** | **49** | **VRKAGIAHLYG-IAG-STNVTGDQVKKLDVLSNDLVINVLKSSFATCVLV** |
|  | ***ec*FBPase** | **40** | **INKAGLVDILG-ASG-AENVQGEVQQKLDLFANEKLKAALKARDIVAGIA** |
|  |  |  | **:.:**: : * : *. *: ***:::* . : : .. :** |
|  |  |  |  |
|  |  |  | **/================//////====\/\/\/===/////////=====** |
|  | ***Lm*FBPase** | **96** | **SEEEEQMIIAESGRRGDYLIFFDPLDGSSNIDANVSVGSIWGVWRLPKDT** |
|  | ***h*lFBPase** | **97** | **SEEDKHAIIVEPEKRGKYVVCFDPLDGSSNIDCLVSVGTIFGIYRK-KST** |
|  | ***h*mFBPase** | **97** | **SEENKDAIITAKEKRGKYVVCFDPLDGSSNIDCLASIGTIFAIYRK-TSE** |
|  | ***p*lFBPase** | **97** | **SEEDKNAIIVEPEKRGKYVVCFDPLDGSSNIDCLVSIGTIFGIYRK-NST** |
|  | ***ec*FBPase** | **88** | **SEEEDEIVVFEGCEHAKYVVLMDPLDGSSNIDVNVSVGTIFSIYRRVTPV** |
|  |  |  | *****:.. :: .:..*:: :********** .*:*:*:.::* .** |
|  |  |  |  |
|  |  |  | **=====\/\/\/\/\/==\/\=////////===//////=====//////=** |
|  | ***Lm*FBPase** | **146** | **TIN-SVEDANAVIRMLKGTDMVSAGYAVYGSATNLVLTSGHGVDGFTLDP** |
|  | ***h*lFBPase** | **146** | **DEP-SEKDA-----LQPGRNLVAAGYALYGSATMLVLAMDCGVNCFMLDP** |
|  | ***h*mFBPase** | **146** | **DEP-SEKDA-----LQCGRNIVAAGYALYGSATLVALSTGQGVDLFMLDP** |
|  | ***p*lFBPase** | **146** | **DEP-SEKDA-----LQPGRNLVAAGYALYGSATMLVLAMVNGVNCFMLDP** |
|  | ***ec*FBPase** | **138** | **GTPVTEEDF-----LQPGNKQVAAGYVVYGSSTMLVYTTGCGVHAFTYDP** |
|  |  |  | **. : * . *:***.:***:* :. : **. * **** |
|  |  |  |  |
|  |  |  | **==///////==========///=====/\/==\/\/\/\/\/\/======** |
|  | ***Lm*FBPase** | **195** | **NIGEFILTHPHISIPKKRSIYSVNEGNYGKWEPWFKEYIDYLKM--NKTT** |
|  | ***h*lFBPase** | **190** | **AIGEFILVDKDVKIKKKGKIYSLNEGYARDFDPAVTEYIQRKKFPPDNSA** |
|  | ***h*mFBPase** | **190** | **ALGEFVLVEKDVKIKKKGKIYSLNEGYAKYFDAATTEYVQKKKFPEDGSA** |
|  | ***p*lFBPase** | **190** | **AIGEFILVDRDVKIKKKGSIYSINEGYAKEFDPAITEYIQRKKFPPDNSA** |
|  | ***ec*FBPase** | **183** | **SLGVFCLCQERMRFPEKGKTYSINEGNYIKFPNGVKKYIKFCQEEDKSTN** |
|  |  |  | **:* * * . : : :* . **:*** : .:*:. : .** |
|  |  |  |  |
|  |  |  | **===////===\/\/\/\/\/\==////================\/\/\/\** |
|  | ***Lm*FBPase** | **243** | **-RYSARYIGSMVGDIHRTLLYGGIFCYPKDANQVEGKLRLLYEAAPMAMI** |
|  | ***h*lFBPase** | **240** | **-PYGARYVGSMVADVHRTLVYGGIFLYPANKKSPNGKLRLLYECNPMAYV** |
|  | ***h*mFBPase** | **240** | **-PYGARYVGSMVADVHRTLVYGGIFLYPANQKSPKGKLRLLYECNPVAYI** |
|  | ***p*lFBPase** | **240** | **-PYGARYVGSMVADVHRTLVYGGIFMYPANKKSPKGKLRLLYECNPMAYV** |
|  | ***ec*FBPase** | **233** | **RPYTSRYIGSLVADFHRNLLKGGIYLYPSTASHPDGKLRLLYECNPMAFL** |
|  |  |  | *** :**:**:*.*.**.*: ***: ** . .********. *:* :** |
|  |  |  |  |
|  |  |  | **\/\/==///=====\/\===========////=/\/\/\/\//\/=** |
|  | ***Lm*FBPase** | **292** | **VEQAGGKAVGSNGRILEQSITRLHQRTPVYFGSRQEVDLCMAFRDRNVKT** |
|  | ***h*lFBPase** | **289** | **MEKAGGMATTGKEAVLDVIPTDIHQRAPVILGSPDDVLEFLKVYEKH---** |
|  | ***h*mFBPase** | **289** | **IEQAGGLATTGTQPVLDVKPEAIHQRVPLILGSPEDVQEYLTCVQKN---** |
|  | ***p*lFBPase** | **289** | **MEKAGGLATTGKEAVLDIVPTDIHQRAPIILGSPEDVTELLEIYQKH---** |
|  | ***ec*FBPase** | **283** | **AEQAGGKASDGKERILDIIPETLHQRRSFFVGNDHMVEDVERFIREF---** |
|  |  |  | ***:*** * .. :*: :*** . .*. . * .** |
|  |  |  |  |
|  | ***Lm*FBPase** | **342** | **EALAPTSSKL** |
|  | ***h*lFBPase** | **336** | **-------SAQ** |
|  | ***h*mFBPase** | **336** | **------QAGS** |
|  | ***p*lFBPase** | **336** | **-------AAK** |
|  | ***ec*FBPase** | **330** | **-------PDA** |
|  |  |  |  |

**Fig. S1.** Structure-based sequence alignment of *Lm*FBPase (O97193), human liver isozyme (*h*lFBPase, P09467), human muscle isozyme (*h*mFBPase, O00757), pig liver isozyme (*p*lFBPase, P00636) and *E. coli* Type I isozyme (*ec*FBPase, P0A993). The secondary structure elements of *Lm*FBPase are shown above the alignment. Helices are shown by red, β-strands are shown by yellow, and loop regions are shown by green. The ‘dynamic loops’ (residues 52–71) are highlighted by purple background. Residues conserved in all aligned FBPases are labelled by an asterisk (*), whereas a colon (:) and a period (.) indicate strongly similar and weakly similar sequences, respectively. Residues at the active sites are highlighted in range. Residues which interact with AMP are highlighted in cyan. The sequence alignment was performed with MUSCLE [1]. Methionines shown as the first amino acids in the sequences correspond to start codons ‘ATG’ in the genes, but there is no experimental evidence that these methionines are present in the mature enzymes to the authors’ best knowledge.

**Table S1.** Matrix of sequence identities of FBPases*^1^*

|  | *Lm*FBPase | *h*lFBPase | *h*mFBPase | *p*lFBPase | *ec*FBPase |
| --- | --- | --- | --- | --- | --- |
| *Lm*FBPase | 100 | 43.5 | 41.3 | 43.2 | 40.4 |
| *h*lFBPase | 43.5 | 100 | 76.6 | 89.9 | 43.6 |
| *h*mFBPase | 41.3 | 76.6 | 100 | 76.0 | 40.8 |
| *p*lFBPase | 43.2 | 89.9 | 76.0 | 100 | 42.1 |
| *ec*FBPase | 40.4 | 43.6 | 40.8 | 42.1 | 100 |

*^1^* Sequence identities of *Lm*FBPase (O97193), human liver isozyme (*h*lFBPase, P09467), human muscle isozyme (*h*mFBPase, O00757), pig liver isozyme (*p*lFBPase, P00636) and *E. coli* Type I isozyme (*ec*FBPase, P0A993) are calculated with Clustal Omega [2].





*K*i[Mn^2+^]=44.3μM

*K*i[Ca^2+^]=31.4μM

**(a)**

**(b)**

*K*_a_[Mg^2+^]=1.2mM

**Fig. S2.** Enzymatic characterization of the effects of metal on *Lm*FBPase activity. A, *Lm*FBPase requires magnesium for activity. B, Mn^2+^ and Ca^2+^ inhibited the activity of *Lm*FBPase in the presence of 10 mM Mg^2+^.


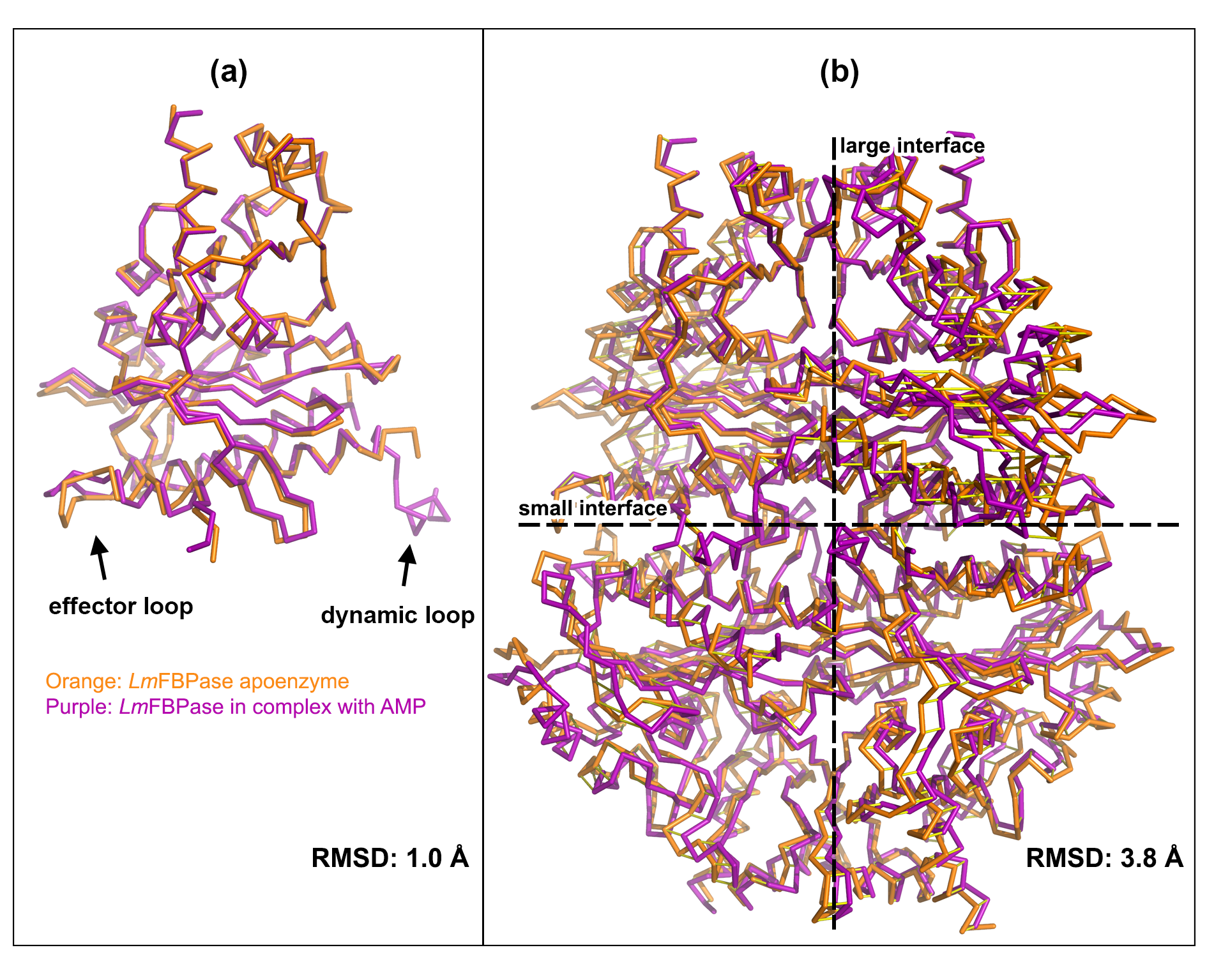


**Fig. S3.** Superposition of *Lm*FBPase structures. (a) Superposition of monomers of *Lm*FBPase apoenzyme structure (orange) and *Lm*FBPase in complex with AMP (purple). Major conformational changes are highlighted with arrows. (b) Overall superposition of *Lm*FBPase apoenzyme structure (orange) and *Lm*FBPase in complex with AMP (purple). Small and large interfaces are shown in dashed lines.


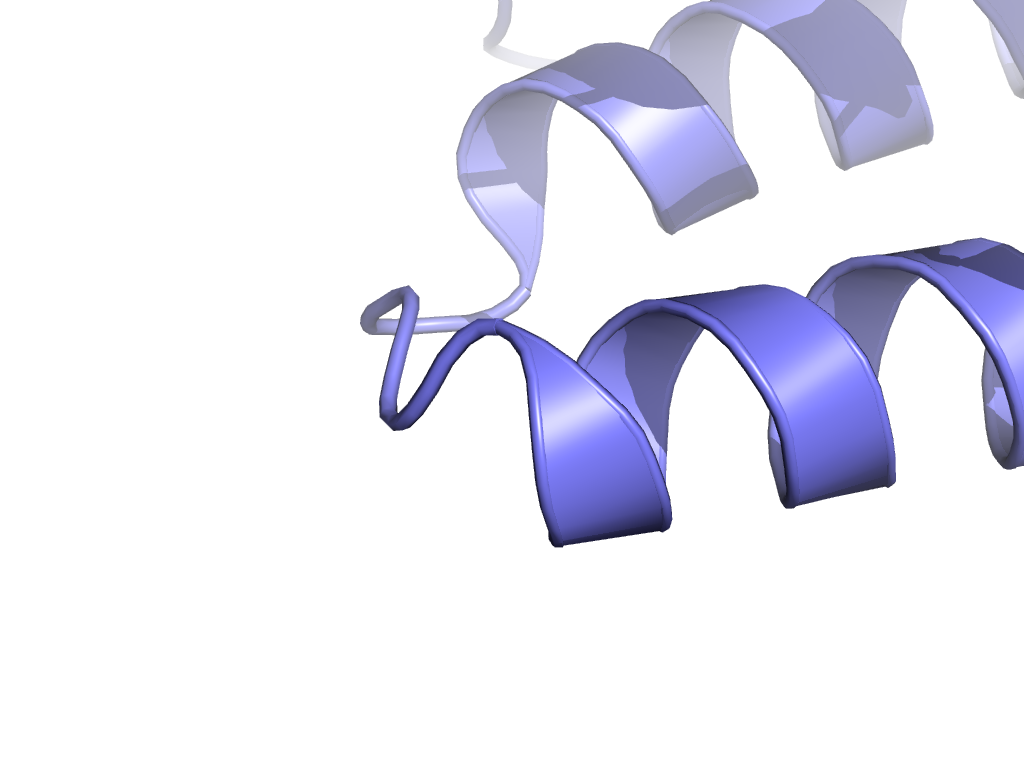

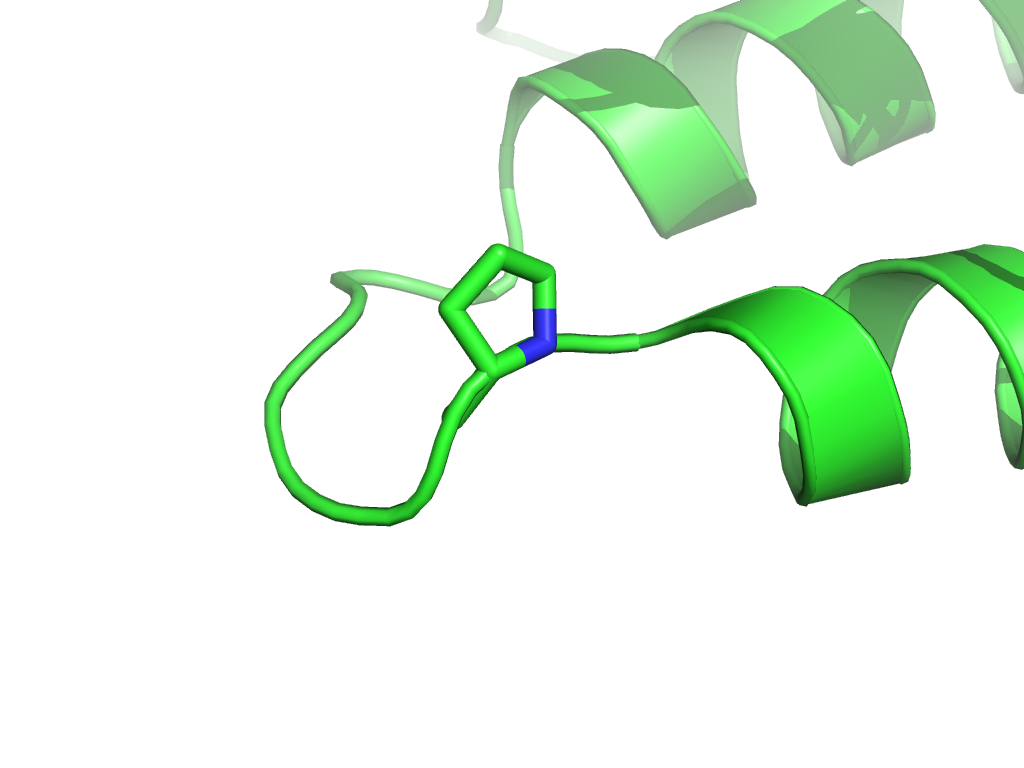


(a) human FBPase

(b) *Leishmania* FBPase

Pro21

**extended loop**

**H1**

**H2**

**H1**

**H2**

**Fig. S4.** Comparison between the effector loop of human and *Leishmania* FBPases. Structure of human liver FBPase (PDB ID: 1FTA) is shown in blue. Structure of *Leishmania* FBPase is shown in green. The species-specific residue Pro21 that breaks the α-helix H1 thereby extending the effector loop is shown in sticks.

Kinetoplastid bodonid Bodo saltans KVVEKNIR**R**AGLH

Kinetoplastid trypanosomatid Phytomonas sp. KVTEKNIR**R**AGMK

Endotrypanum monterogei KVIEKNIR**R**AGMK

Leishmania tarentolae KVIEKNIR**R**AGMK

Leishmania braziliensis KVIEKNIR**R**AGMK

Leishmania panamensis KVIEKNIR**R**AGMK

Leishmania mexicana KVIEKNIR**R**AGMK

Leishmania major KVIEKNIR**R**AGMK

Leishmania donovani KVIEKNIR**R**AGMK

Crithidia fasciculata KVIEKNIR**R**AGMK

Leptomonas pyrrhocoris KLIEKNIR**R**AGMK

Leptomonas seymouri KLIEKNIR**R**AGMK

American Trypanosoma cruzi CL Brener KVIEKHIR**S**AGMQ

Trypanosoma rangeli KVIEKHIR**S**AGMQ

Trypanosoma grayi KVIEKNIR**S**AGMQ

African Trypanosoma vivax KVVERNIR**T**AGMQ

Trypanosoma congolense KVVEMNIR**S**AGAQ

Trypanosoma gambiense KVIEMNIR**S**AGAQ

Trypanosoma equiperdum KVIEMNIR**S**AGAQ

Trypanosoma brucei TREU927 KVIEMNIR**S**AGAQ

Trypanosoma brucei 427 KVIEMNIR**S**AGAQ

Prokaryotes Anabaena variabilis KLVARRLS**R**AGLM

Escherichia coli KIIHRDIN**K**AGLV

Fungi Saccharomyces cerevisiae KFVSHTIR**R**AELV

Schizosaccharomyces pombe KFIANTIR**K**AELV

Mammals human KAISSAVR**K**AGIA

Plants rice KFVASAVN**K**AGLA

**Fig. S5.** Multiple alignment of the segment of FBPase sequences from different organisms around the residue corresponding to Arg48 in the *L. major* enzyme (residue highlighted in colour). Sequences were retrieved from the NCBI (<https://www.ncbi.nlm.nih.gov)> and TriTrypDB (<http://tritrypdb.org/tritrypdb/)> databases. All kinetoplastid sequences (except that of Bodo saltans of which the sequence in the database seems incomplete) contain a C-terminal peroxisomal-targeting signal (not shown), indicating that the enzyme functions within the glycosomes of these organisms.


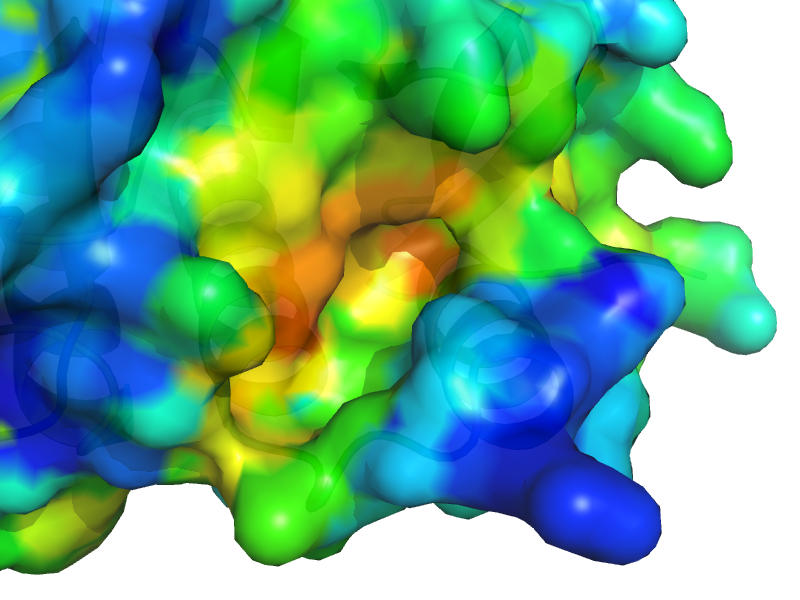

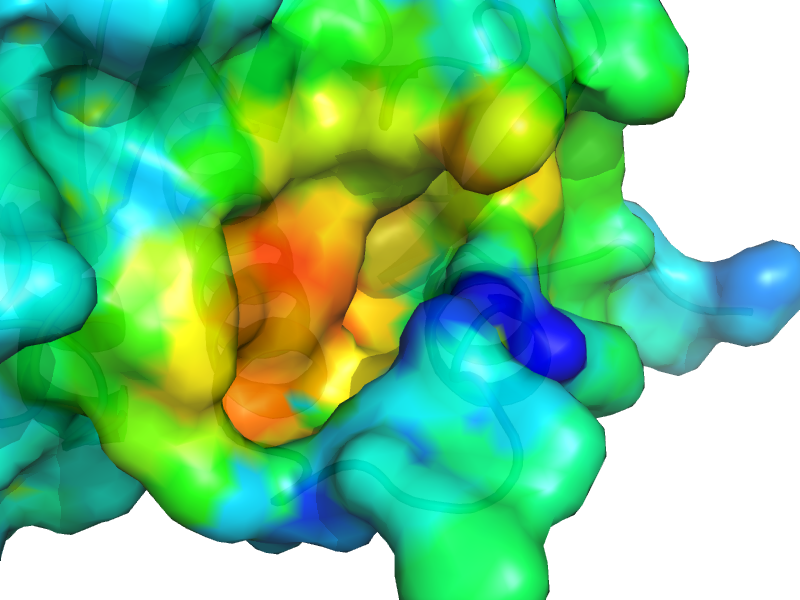


**(a) human liver FBPase**

**(b) *Leishmania* FBPase**

**effector site**

**effector site**

**Druggability scores of the effector site of human liver FBPase**

| **Program** | **Score** |
| --- | --- |
| DoGSiteScorer | 0.60 |
| FPocket | 13 |
| STP | colored in the left figure |

**Druggability scores of the effector site of *Leishmania* FBPase**

| **Program** | **Score** |
| --- | --- |
| DoGSiteScorer | 0.84 |
| FPocket | 13 |
| STP | colored in the left figure |

**Fig. S6.** Evaluation of the ‘druggability’ of the effector pockets of human liver FBPase and *Leishmania* FBPase. In the figures on the left, as predicted by STP [3], the ligand-binding favourable positions in the protein are shown in red, while blue represents ligand-binding unfavorable parts. Druggability scores predicted using other programs DoGSiteScorer [4] and FPocket [5] are shown on the tables on the right. Crystal structures of human liver FBPase (PDB ID: 1FTA) and *Lm*FBPase in complex with AMP were used for the predictions.

**References for Supplementary Data**

[1] Edgar, R. C. (2004). MUSCLE: a multiple sequence alignment method with reduced time and space complexity. BMC Bioinformatics. 5, 113.

[2] Sievers, F., Wilm, A., Dineen, D., Gibson, T. J., Karplus, K., Li, W. Z., Lopez, R., McWilliam, H., Remmert, M., Soding, J., Thompson, J. D. & Higgins, D. G. (2011). Fast, scalable generation of high-quality protein multiple sequence alignments using Clustal Omega. Mol. Syst. Biol. 7, 1-6.

[3] Mehio, W., Kemp, G. J., Taylor, P. & Walkinshaw, M. D. (2010). Identification of protein binding surfaces using surface triplet propensities. Bioinformatics. 26, 2549-2555.

[4] Volkamer, A., Kuhn, D., Rippmann, F. & Rarey, M. (2012). DoGSiteScorer: a web server for automatic binding site prediction, analysis and druggability assessment. Bioinformatics. 28, 2074-2075.

[5] Le Guilloux, V., Schmidtke, P. & Tuffery, P. (2009). Fpocket: an open source platform for ligand pocket detection. BMC Bioinformatics. 10, 168.
