# Supplementary figures and images for: Structures of Leishmania Fructose-1,6-Bisphosphatase Reveal Species-Specific Differences in the Mechanism of Allosteric Inhibition
Source: J Mol Biol. 2017 Oct 13;429(20):3075–89. doi: 10.1016/j.jmb.2017.08.010 (PMC5639204; doi:10.1016/j.jmb.2017.08.010)

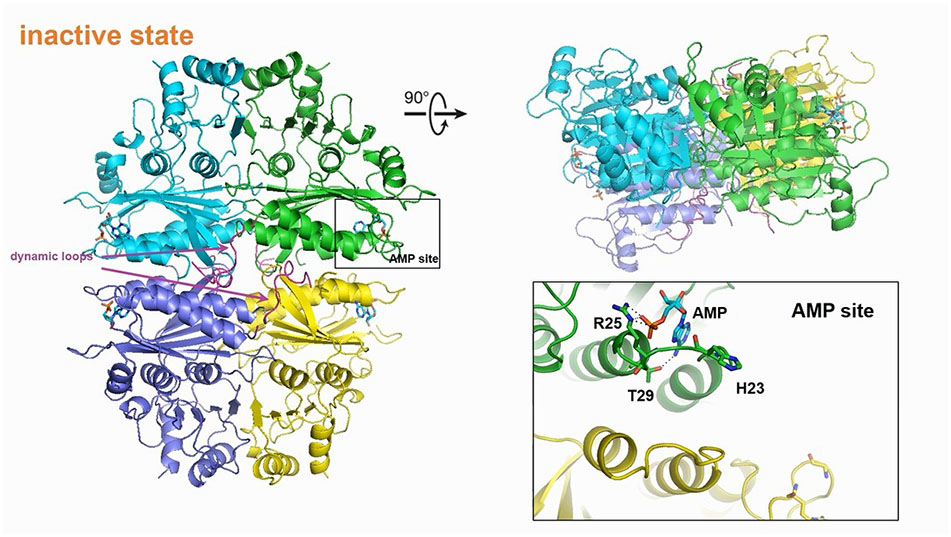

Supplement: Supplementary movie — Animation of structural transitions between T- and R-states of Leishmania Fructose-1,6-Bisphosphate. [file mmc2.jpg]
